# Structure and Photoluminescence of WO_3-*x*_ Aggregates Tuned by Surfactants

**DOI:** 10.3390/mi13122075

**Published:** 2022-11-25

**Authors:** Biben Wang, Xiaoxia Zhong, Haiyan Xu, Yongcai Zhang, Uros Cvelbar, Kostya (Ken) Ostrikov

**Affiliations:** 1 College of Chemistry and Chemical Engineering, Chongqing University of Technology, 69 Hongguang Rd, Lijiatuo, Banan District, Chongqing 400054, China; 2 State Key Laboratory of Advanced Optical Communication and Networks, Key Laboratory for Laser Plasmas (Ministry of Education), School of Physics and Astronomy, Shanghai Jiao Tong University, Shanghai 200240, China; 3 School of Materials and Chemical Engineering, Anhui Jianzhu University, Hefei 230601, China; 4 School of Chemistry and Chemical Engineering, Yangzhou University, Yangzhou 225009, China; 5 Jozef Stefan Institute, Jamova Cesta 39, SI-1000 Ljubljana, Slovenia; 6 School of Chemistry and Physics, Queensland University of Technology, Brisbane, QLD 4000, Australia; 7 Centre for Materials Science, Queensland University of Technology, Brisbane, QLD 4000, Australia

**Keywords:** WO_3-*x*_ nanocrystals, steric repulsion, aggregation, surfactants, photoluminescence

## Abstract

The optoelectronic properties of transition metal oxide semiconductors depend on their oxygen vacancies, nanostructures and aggregation states. Here, we report the synthesis and photoluminescence (PL) properties of substoichiometric tungsten oxide (WO_3-*x*_) aggregates with the nanorods, nanoflakes, submicro-spherical-like, submicro-spherical and micro-spherical structures in the acetic acid solution without and with the special surfactants (butyric or oleic acids). Based on theory on the osmotic potential of polymers, we demonstrate the structural change of the WO_3-*x*_ aggregates, which is related to the change of steric repulsion caused by the surfactant layers, adsorption and deformation of the surfactant molecules on the WO_3-*x*_ nanocrystals. The WO_3-*x*_ aggregates generate multi-color light, including ultraviolet, blue, green, red and near-infrared light caused by the inter-band transition and defect level-specific transition as well as the relaxation of polarons. Compared to the nanorod and nanoflake WO_3-*x*_ aggregates, the PL quenching of the submicro-spherical-like, submicro-spherical and micro-spherical WO_3-*x*_ aggregates is associated with the coupling between the WO_3-*x*_ nanoparticles and the trapping centers arising from the surfactant molecules adsorbed on the WO_3-*x*_ nanoparticles.

## 1. Introduction

Tungsten oxides are important semiconductor materials. Since their excellent physical and chemical properties are linked to the size, oxygen vacancies, nanostructures and aggregation states, it is still a significant challenge to tune their structures and properties [1,2]. When inorganic nanomaterials are synthesized in solutions, surfactants are universally used to tune their structures by adjusting the aggregation of nanocrystals [3,4,5,6]. For example, the In_2_O_3_ nanodots are aggregated into nanoflowers depending on myristic acid [6]. Furthermore, the studies indicate that some properties of the aggregated materials (e.g., carrier transport and photoluminescence) strongly depend on the coupling separation between the nanocrystals [5,7], which are related to the lengths of the surfactant molecules [8,9,10]. In other words, surfactants play important roles in tuning the aggregated nanomaterials’ structures and properties. In a solution containing the surfactants, there are attraction and repulsive interactions between the nanocrystals. The attraction interactions (i.e., dipole-dipole and van der Waals interactions) promote the aggregation between the nanocrystals, while the repulsive interactions (e.g., steric and electrostatic repulsion forces) hinder the aggregation [3,11]. Due to the strong attraction interactions, the nanocrystals are aggregated together [11]. Therefore, tuning of the aggregation process weakens the attractions by the introduction of the surfactants. This paper explores the tuning of the structures and photoluminescence (PL) properties of substoichiometric tungsten oxide (WO_3-*x*_, 2 < *x* < 3) aggregates through adjusting the aggregation process using the solvothermal synthesis technique.

Compared to stoichiometric tungsten oxides (WO_3_ and WO_2_), the WO_3-*x*_ nanomaterials have fascinating luminescent and catalytic properties, which are related to the aggregation and the defect levels formed by the oxygen vacancies [12,13,14,15,16,17]. In other words, the WO_3-*x*_ materials are becoming the important semiconductor materials for optoelectronic and catalytic applications. Therefore, the WO_3-*x*_ materials have attracted considerable attention and great efforts contribute to the foundation and application studies in recent years [14,15,17,18,19,20,21].

Various WO_3-*x*_ nanostructures (e.g., films, nanowires, nanodots and hybrid nanostructures) have been fabricated using physical and chemical methods [12,14,19,20,21]. In particular, the WO_3-*x*_ nanomaterials are universally synthesized in different solvents using the solvothermal method [22]. It is well known that various WO_3-*x*_ nanostructures are made up of nanoparticles, i.e., the WO_3-*x*_ nanostructures can be tuned by adjusting the aggregation of the WO_3-*x*_ nanoparticles. The early studies indicate that the aggregation of the WO_3-*x*_ nanoparticles is indeed tuned by the surfactants [23,24]. However, the mechanism has not been well understood because the repulsion effects caused by the surfactants involve the lengths, adsorption, compress, etc. of the surfactant molecules [25]. In this paper, the special surfactants (i.e., butyric acid and oleic acid) were employed to tune the structures of the WO_3-*x*_ aggregates, respectively. Based on the osmotic potential of polymers, the effects of the surfactants on the aggregation of the WO_3-*x*_ nanoparticles are analyzed. Furthermore, the PL properties of the WO_3-*x*_ aggregates were studied.

## 2. Materials and Method

### 2.1. Materials

In the process of synthesizing the WO_3-*x*_ aggregates, the WCl_6_ powder, acetic, butyric and oleic acids were used as the precursors. Among these precursors, the WCl_6_ powder (99%) was supplied by Yanyu (Shanghai) Chemical Agent Co., Ltd.) and all the carboxylic acids including acetic acid (≥99.8%), butyric acid (99%) and oleic acid (chemical grade) were provided by Shanghai Macklin Biochemical Technology Co., Ltd. (Shanghai, China). The previous studies indicate that the metal acetate was used to fabricate the corresponding metal oxide nanocrystals [6]. Here, the WCl_6_ powder and acetic acid was used to synthesize the WO_3-*x*_ nanocrystals, and the butyric and oleic acids were used as the surfactants to tune the structure of the WO_3-*x*_ aggregates, respectively.

### 2.2. Synthesis

Due to differences in the viscosity of different carboxylic acids, the acetic acid and surfactant were mixed in a 100 mL Teflon-lined autoclave by magnetic stirring for 30 min to ensure the uniformity of the solution. After the WCl_6_ powder was poured into the solution, the solution was further stirred magnetically for 30 min, and then the autoclave was encapsulated in a stainless tank. In a heating furnace, the tank was heated to 120 °C in ~60 min and maintained for 12 h at this temperature. The loose WO_3-*x*_ agglomerate was obtained at room temperature by centrifugal precipitation and drying treatment for 12 h at 50 °C. Furthermore, the agglomerate was ground into the powder to use for further characterizations. Different WO_3-*x*_ samples were synthesized in the experiments and the synthesis conditions are summarized in Table 1.

### 2.3. Characterization and PL Measurement

The phases and composition of the as-synthesized WO_3-*x*_ aggregates are determined according to the data recorded in a TTRAX3 X-ray diffractometer (XRD) using a Cu K*α* radiation with a wavelength of 0.154 nm and a Horiba Scientific LabRAM HR evolution Raman spectrometer under irradiation of the 532 nm line of a semiconductor laser, respectively. The binding states of elements were analyzed depending on the data obtained from an ESCALAB 250 X-ray photoelectron spectroscopy (XPS) equipped with an Al K*α* X-ray source. The studies on the surface morphology and microstructures were carried out in an S-4800 field emission scanning electron microscope (FESEM) (operated at 5 kV) and a TECNAI G^2^ F30 high-resolution transmission electron microscope (TEM) (operated at 300 kV), respectively.

At room temperature, the PL emission from the as-synthesized aggregates was measured in the Horiba Scientific LabRAM HR evolution Raman spectrometer under irradiation of the He-Cd laser with a wave length of 325 nm and ~1 μm diameter of laser beam. During the PL measurement, the power of laser and the acquisition time were set to 6.8 mW and 30 s, respectively.

## 3. Results

### 3.1. Phases and Composition

**Figure 1:**
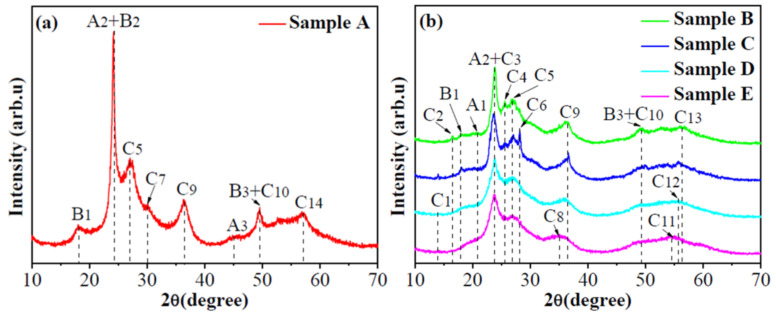
XRD patterns of as-synthesized WO_3-*x*_ aggregates of (**a**) sample A and (**b**) samples B–E respectively, which indicate that they are composed of mixed phases.

The phases and composition of the as-synthesized WO_3-*x*_ aggregates are determined by the XRD and Raman results. The XRD patterns of samples A–D are presented in Figure 1 and the phases corresponding to the peaks are summarized in Table 2. According to the data in Table 2, the as-synthesized materials are the WO_3-*x*_ materials composed of the W_19_O_55_, WO_2.92_ and W_18_O_49_ phases. Furthermore, Figure 1 indicates that the strongest peaks in Figure 1b become wide compared to the strongest peak in Figure 1a. In other words, the size of WO_3-*x*_ nanoparticles synthesized in the solution containing the surfactant becomes small.

**Figure 2:**
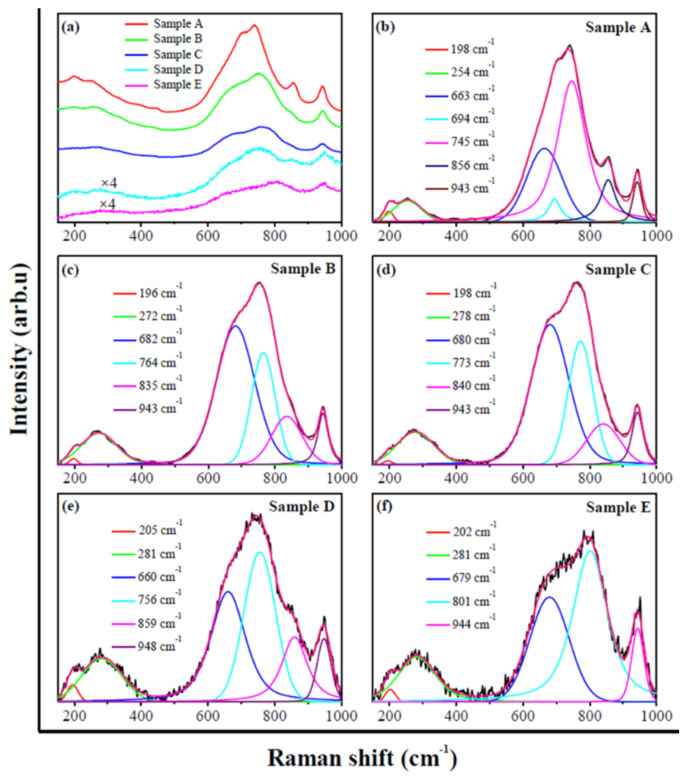
Raman spectra (**a**) and deconvoluted Raman spectra (**b**–**f**) of as-synthesized WO_3-*x*_ aggregates. The data in the insets are the positions corresponding to Raman peaks.

The Raman spectra presented in Figure 2 further evidence that the as-synthesized materials are the WO_3-*x*_ aggregates. To clearly distinguish the Raman peaks, the Raman spectra in Figure 2a are deconvoluted and the results are presented in Figure 2b−f. In Table 3, the deconvoluted Raman peaks are assigned and summarized. Depending on the studies [26], the Raman peaks at 835−859 cm^−1^ may result from the vibration modes of different W−O bonds. 

Furthermore, the Raman peaks in a range of 200−400 cm^−1^ are associated with the vibration of W^5+^−O bonds, which indicate the formation of the WO_3-*x*_ aggregates [29]. In addition, the studies indicate that the Raman spectrum of pure WO_3_ shows the sharp characteristic peaks at about 806, 716, 326 and 272 cm^−1^, while the spectra of WO_3-*x*_ exhibit the wide peaks at these positions and some new peaks due to the distortions in the crystalline structure [30]. According to the studies [30], the Raman peaks in Figure 2 further address that the as-synthesized materials are the WO_3-*x*_ aggregates.

### 3.2. Binding States of Elements

**Figure 3:**
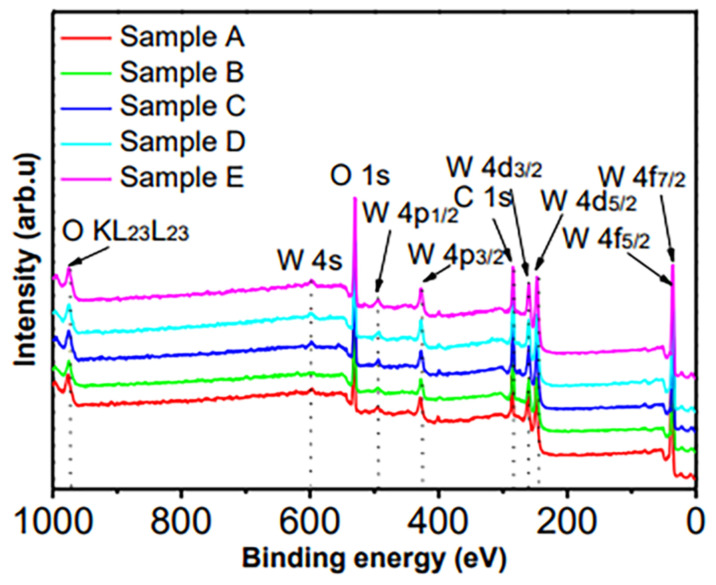
XPS survey spectra of samples A−D. The XPS peaks are assigned according to Ref. [31]. These XPS peaks indicate that the as-synthesized aggregates contain the W, O and C elements.

**Figure 4:**
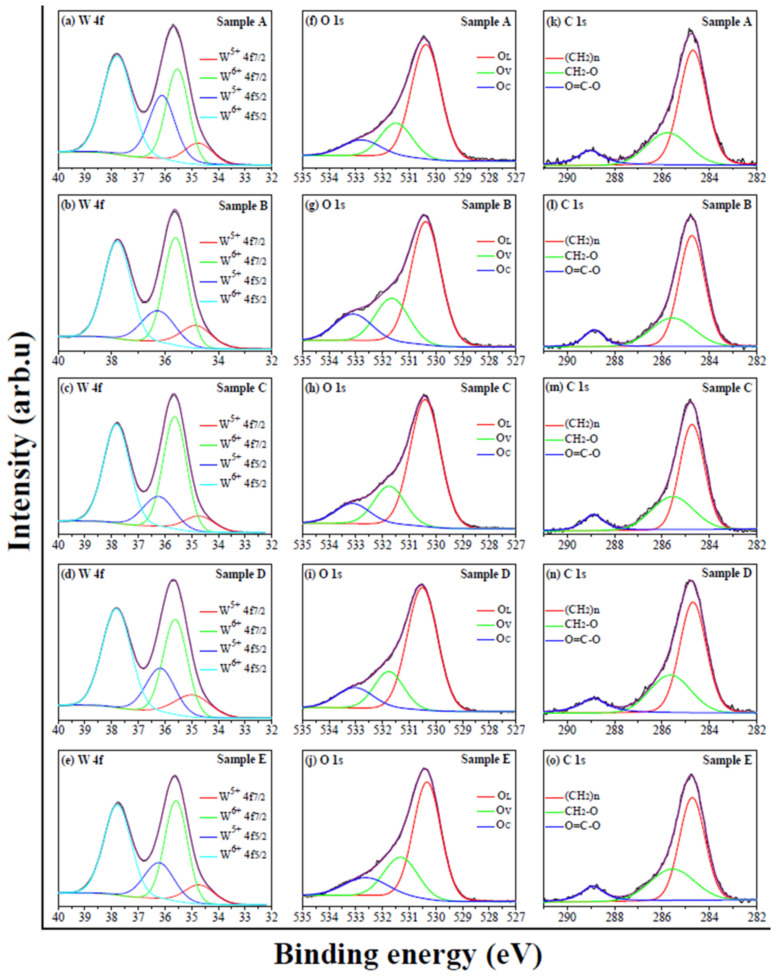
High-resolution W 4f, O 1s and C 1s XPS spectra of as-synthesized WO_3-*x*_ aggregates. The deconvoluted peaks indicate the presence of oxygen vacancies and the absorption of carboxylic acid molecules.

The XPS spectra are usually used to analyze the binding states of elements. The XPS survey spectra presented in Figure 3 indicate that the as-synthesized aggregates contain the W, O and C elements. In order to confirm the binding states of these elements, a XPSPEAK 41 analysis software was used to deconvolute the W 4f, O 1s and C 1s XPS spectra after Shirley background subtraction. The results are presented in Figure 4 and the deconvoluted peaks are summarized in Table 4.

From Figure 4a–d, one can see that the W 4f XPS spectra feature two pair of peaks, i.e., one pair of peaks at about 34.7−34.9 eV and 36.1−36.3 eV are attributed to W^5+^ 4f_7/2_ and W^5+^ 4f_5/2_, and another pair at about 35.5−35.6 eV and 37.8 eV correspond to W^6+^ 4f_7/2_ and W^6+^ 4f_5/2_ [32,33]. The O 1s XPS spectra in Figure 4f−j are deconvoluted into three peaks located at about 530.3−530.5, 531.3−531.7 and 532.7−533.1 eV, which are related to the oxygen atoms in the lattices (O_L_), the oxygen in the vicinity of oxygen vacancies (O_V_) and the oxygen atoms of carboxylic groups (O_C_) [32,34], respectively. Similarly, the C 1s XPS spectra in Figure 4k–o are fitted by three peaks at about 284.7, 285.5−285.8 and 288.9−289.0 eV, and they are attributed to (CH_2_)*n*, CH_2_-O and O=C-O [31,34], respectively.

The appearance of the XPS peaks related to W^5+^ indicates the formation of the WO_3-*x*_ structure and the creation of oxygen vacancies [16]. Furthermore, the O 1s XPS spectra confirm the presence of oxygen vacancies, and the carboxyl acid molecules are adsorbed on the surfaces of the WO_3-*x*_ aggregates and they are bonded on the surface in the bidentate interaction modes [35].

**Figure 5:**
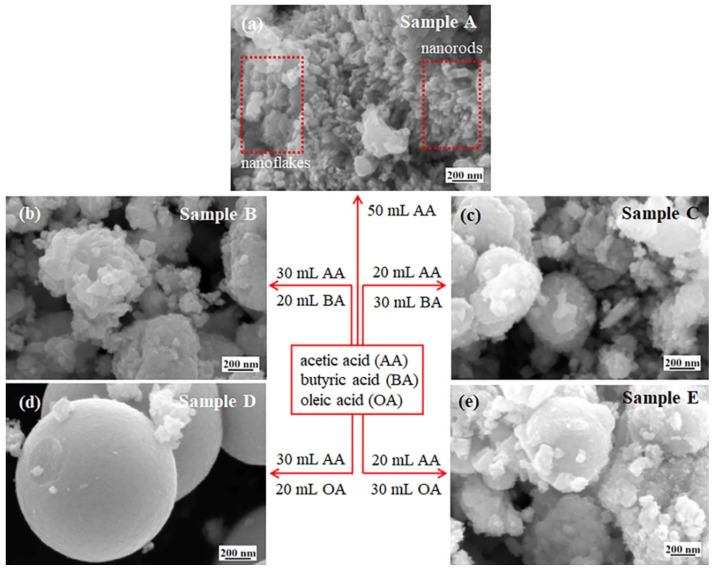
FESEM images of samples A−E. These images indicate that the as-synthesized WO_3-*x*_ materials are the mixture of (**a**) nanoflakes and aggregated nanodots, (**b**,**e**) the submicro-spherical-like, (**c**) submicro-spherical and (**d**) micro-spherical structures mixed with the aggregated nanoparticles.

According to Figure 4f–j, the areas of O_L_, O_V_ and O_C_ peaks are obtained and they are summarized in Table 5. Referring to Ref. [36], the coverage ratios of the oxygen vacancies on the sample surfaces (i.e., the area ratio R of O_V_ peak to the area sum of O_L_ and O_V_ peaks) are calculated and the results are shown in Table 5. The studies indicate that the insulativity of metal oxides is enhanced by the reduction of oxygen vacancies [37]. In other words, the conductivity is enhanced by the oxygen vacancies. Based on the relation of the conductivity with the oxygen vacancies, the data in Table 5 indicate that the conductivity of sample B is high compared to sample A. Furthermore, the conductivity of samples C and D reduces compared to sample B. However, the conductivity of sample E becomes higher than sample B. The change in the conductivity may originate from the structural conversion of the WO_3-*x*_ aggregates and the amount of carboxylic acid as well as the lengths of carboxylic acid molecules. The SEM images (see Figure 5) indicate that the structures of samples A and B convert from the nanorods and nanoflakes to the aggregates of the nanoparticles. As a result, the surface area of sample B is enhanced to increase the oxygen vacancies. Compared to sample B, Table 1 indicates that the amount of butyric acid increases for sample C, which implies that more butyric acid molecules adsorb on the surface of the WO_3-*x*_ nanoparticles to increase the insulativity. Therefore, the conductivity is reduced. When sample D was synthesized, oleic acid was employed. Due to the long chain of an oleic acid molecule, the insulativity of sample D is enhanced to reduce the conductivity. For sample E, much oleic acid was used. The high viscosity of oleic acid leads to the reduction of the oleic acid molecules adsorbed on the WO_3-*x*_ nanoparticles (see the analyses in Section 3.4). As a consequence, the conductivity is increased.

### 3.3. Morphology and Microstructure

The morphologies of the as-synthesized WO_3-*x*_ aggregates are presented in Figure 5. Figure 5a exhibits that sample A contains the nanoflakes and nanorods as well as some aggregated nanoparticles, which indicate that the WO_3-*x*_ nanoparticles are aggregated in an oriented mode in acetic acid solution.

Figure 5b−e exhibit the mixture of the submicro-spherical-like, submicro-spherical and micro-spherical structures mixed with the aggregated nanoparticles, respectively. These indicate that the aggregation of the WO_3-*x*_ nanoparticles is changed by the butyric acid or oleic acid, i.e., they are aggregated in a disoriented mode, but the aggregation is related to the amount of the surfactants. From Figure 5b−e, one can see that the increase in the amount of butyric acid leas to the structural conversion from submicro-spherical-like to submicro-spherical shapes, while the enhancement of oleic acid results in the change from micro-spherical to submicro-spherical-like shapes.

**Figure 6:**
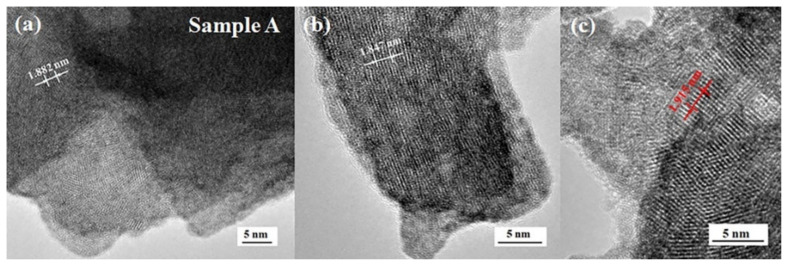
TEM images of sample A, which indicate that the nanoflake (**a**), nanorod (**b**) and nanoparticle (**c**) are the crystal structure with the lattice spacing of ~0.376, 0.369 and 0.383 nm, respectively.

**Figure 7:**
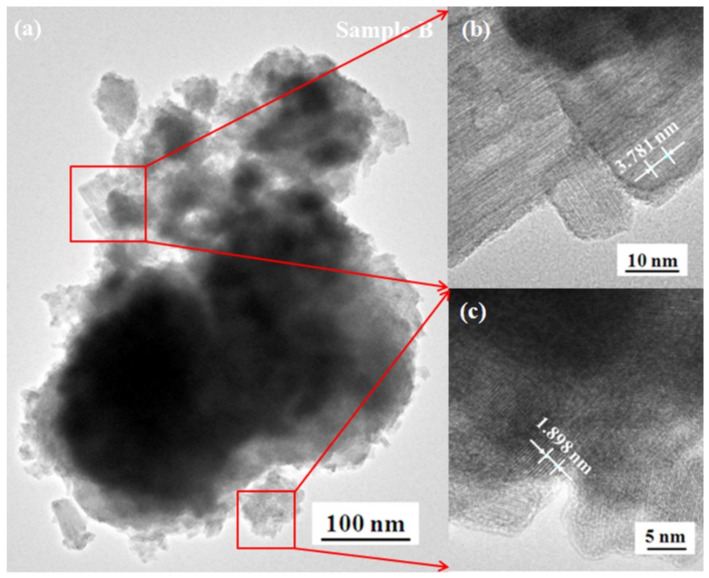
TEM images of sample B. The image (**a**) indicates that sample B is the submicro-spherical-like structure mixed some nanorods and the submicro-spherical-like structure is aggregated by nanoparticles. The high-resolution TEM images indicate that the nanorod (**b**) and nanoparticle (**c**) are the crystalline structure with the lattice spacing of ~0.378 and 0.380 nm, respectively.

**Figure 8:**
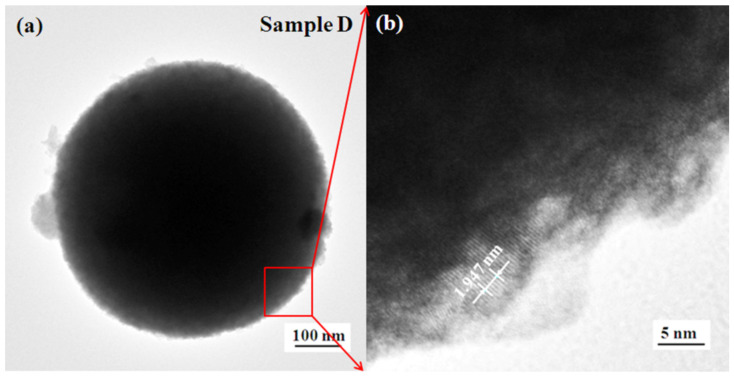
TEM images of sample D. Image (**a**) indicates that the edge is composed of nanoparticles. The high-resolution TEM image (**b**) indicates that the nanoparticle is the crystalline structure with a lattice spacing of ~0.389 nm.

The microstructures of samples A, B and D are revealed in Figure 6, Figure 7, Figure 8. The high-resolution TEM images presented in Figure 6 indicate that the nanoflake, nanorod and nanoparticle in sample A are well crystalized. After the thickness of five crystal planes are measured using digital micrograph software, the lattice distances of the nanoflake, nanorod and nanoparticle are obtained. They are ~0.376, 0.369 and 0.383 nm, respectively, which correspond to the *d* values (0.368 and 0.370 nm) of the WO_2.92_(-1016) and W_19_O_55_(302) [JCPDS: 30-1387 and 45-0167].

The TEM images in Figure 7 indicate that the WO_3-*x*_ aggregates in sample B are the submicro-spherical-like structure mixed with some nanorods. Similar to Figure 6, the thickness of ten or five crystal planes are measured using digital micrograph software. According to the thickness values, the lattice spacing of the nanorod and nanoparticle are, respectively, ~0.378 and 0.380 nm, which correspond to the *d* values (0. 370 and 0.373 nm) of the W_19_O_55_(302) and W_18_O_49_(103) [JCPDS: 45-0167 and 05-0392].

The TEM images of sample D presented in Figure 8 exhibit a micro-spherical structure. According to the measured thickness of five crystal planes, the lattice spacing is about 0.389 nm, which corresponds to the *d* value (0.373 nm) of W_18_O_49_(103) [JCPDS: 05-0392].

### 3.4. Aggregation of WO_3-x_ Nanoparticles in Different Solutions

#### 3.4.1. Aggregation of WO_3-*x*_ Nanoparticles in Acetic Acid

In an earlier paper [13], the formation of the WO_3-*x*_ nanoparticles in acetic acid was studied, which involves the WCl_6_−CH_3_CO_2_H reaction and the condensation reaction. The WCl_6_−CH_3_CO_2_H reaction leads to the formation of tungsten acetate, and the further condensation reaction of tungsten acetate forms the WO_3-*x*_ nanoparticles. In acetic acid solution, the WO_3-*x*_ nanoparticles are covered by the acetic acid molecules. As a result, the WO_3-*x*_ nanoparticles experience a quenching for a long time to form the WO_3-*x*_ nanocrystals (NCs). The XPS results indicate the adsorption of the acetic acid molecules on the surfaces of the WO_3-*x*_ NCs, which indicates that a protecting layer is formed to prevent the aggregation of the WO_3-*x*_ NCs [38]. However, the adsorption is reversible due to the low molecular weight of acetic acid [25,39]. In other words, some acetic acid molecules can be desorbed from the WO_3-*x*_ NCs. Due to the anisotropy of the WO_3-*x*_ NCs, the acetic acid molecules coordinated with the tungsten atoms are changed to selectively adsorb on the special crystal planes [40]. As a result, a strong dipole−dipole interaction occurs between the WO_3-*x*_ NCs. The directionality of the dipole−dipole interaction leads to the aggregation of the WO_3-*x*_ NCs along a direction to form the nanorods [11]. As for the formation of the WO_3-*x*_ nanoflakes, it is related to the gas-liquid interface formed by acetic acid. Due to Brownian motion, some WO_3-*x*_ NCs move to the gas–liquid interface. Some acetic acid molecules adsorbed on the upper and side surfaces of the WO_3-*x*_ NCs on the interface are desorbed. As a result, the strong dipole−dipole interaction between the WO_3-*x*_ NPs leads to the aggregation [11]. During the aggregation, the WO_3-*x*_ NCs are still constrained on the interface. Finally, the aggregation results in the formation of the WO_3-*x*_ nanoflakes.

#### 3.4.2. Aggregation under Surfactants

After the surfactant (i.e., butyric acid or oleic acid) is added, it leads to the WO_3-*x*_ nanoparticles’ size, motion and distribution changes. At the same time, the addition of the surfactant results in the change of the viscosity of the solution. These changes further cause the change in the aggregation process of the WO_3-*x*_ nanoparticles.

1. Changes in size, motion and distribution of WO_3-*x*_ nanoparticles

In the nucleation process, the surfactant molecules are adsorbed on the surfaces of the WO_3-*x*_ nuclei. Unlike the acetic acid molecules, the surfactant molecules have a high molecular wight, which lead to a relatively irreversible adsorption [25,39]. As a result, the nucleation rate is enhanced according to Gibbs equation and energy barrier for nucleation described below. The Gibbs equation is expressed by:

(1) $$d\gamma =-\sum_{i}\Gamma_{i}d\mu_{i}$$

where γ, $\Gamma_{i}$ and $\mu_{i}$ represent the surface tension, the superficial adsorption concentration and chemical potential of the adsorbed species *i* [41], respectively. The nucleation energy barrier $\Delta G_{\mathrm{crit}}$ is expressed by:

(2) $$\Delta G_{\mathrm{crit}}=\frac{16\pi }{3}\frac{\gamma^{3}V_{m}^{2}}{{\left(RT\ln S\right)}^{2}}$$

where R, T, S and $V_{m}$ are universal gas constant, temperature, the supersaturation and the molar volume of monomers [41], respectively. Based on Equations (1) and (2), the adsorption of the surfactant molecules reduces the surface tension, which further lowers the nucleation energy barrier and enhances the nucleation rate. At the same time, the surfactants molecules adsorbed on the surfaces of the WO_3-*x*_ nuclei form the surfactant layers to prevent their aggregation and growth. After these nuclei experience quenching in the solution, they are formed into irregular crystal nanodots (CNDs) with a small size, which are evidenced by the TEM results. Here, we should note that the size refers to the size of the WO_3-*x*_ CNDs without the surfactant layers. Due to the attachment of the surfactant layers on the WO_3-*x*_ CNDs, the size of the WO_3-*x*_ CNDs becomes relatively large due to the long carbon chain of the surfactant molecules. In addition, the surfactants result in the increase in the viscosity η of the solution, which can be addressed by the early studies [42]. In Ref. [42], the results indicate that the viscosity of a mixture composed of an organic compound with high viscosity (e.g., ethanol, butanol or octanol) and another organic compound with low viscosity (e.g., propylamine) reduces with the increase in the mole fraction of the compound with low viscosity. In other words, the viscosity of the mixtures increases with the increase in the mole fraction of the compound with high viscosity. Due to the relation of mole fraction x with volume v (x=ρv/M, where ρ and M are the density and molar mass), the viscosity of the mixtures increases with the increase in the volume of the compound with high viscosity. The viscosity of acetic, butyric and oleic acids at 20 °C is 1.23, 1.54 and 34.8 mPa·s [43,44], respectively. According to these data, the viscosity of the solutions increases with the increase in the volume of the surfactants. Therefore, the introduction of the surfactant leads to the change in the motion of the WO_3-*x*_ CNDs. The relation of diffusion coefficient $D_{\mathrm{diff}}$ with the radius of particle r and viscosity η is:

(3) $$D_{\mathrm{diff}}=\frac{k_{B}T}{6\pi r\eta }$$

where $k_{B}$ is Boltzmann’s constant [22]. According to Equation (3), the diffusion coefficient is reversely proportional to the radius of particle and the viscosity of solution. Therefore, the motion of the WO_3-*x*_ CNDs becomes slow in the solution. In other words, the aggregation of the WO_3-*x*_ CNDs takes place in the interior of the solution. Because the formation of the WO_3-*x*_ CNDs originates from the WCl_6_−CH_3_COO reaction, the slow speed of the WO_3-*x*_ CNDs makes them located in the reaction region, i.e., their distribution in the solution is nonuniform.

2. Aggregation of WO_3-*x*_ nanoparticles

Unlike the aggregation of the WO_3-*x*_ NCs in acetic acid, the WO_3-*x*_ CNDs are aggregated into the submicro-spherical-like, submicro-spherical and micro-spherical structures under the surfactants (Figure 5), which are related to the repulsion interactions between the WO_3-*x*_ CNDs and the property of solution. In this section, the aggregation under the surfactants is analyzed.

**Figure 9:**
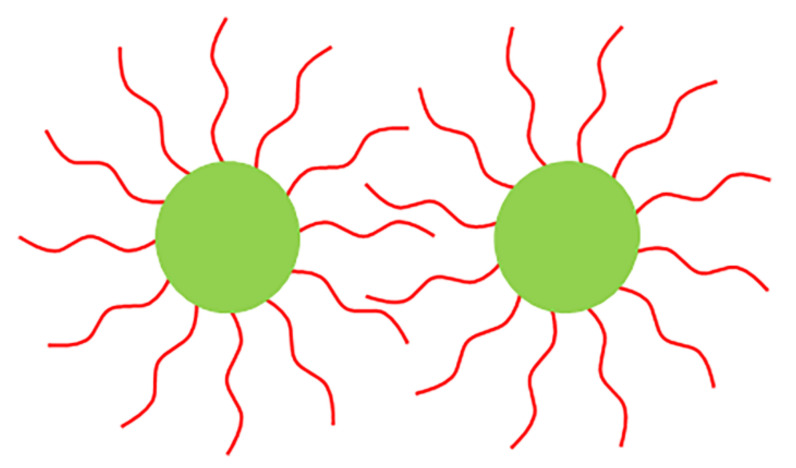
Schematic diagram of steric repulsion produced by the surfactant layers on the WO_3-*x*_ CNDs. Adapted with modification from Refs. [25,45].

For the convenience of analysis, the WO_3-*x*_ CNDs are considered to be a spherical structure and the surfactant molecule is represented by a bending line, which are presented in Figure 9. Due to Brownian motion, the surfactant layers are overlapped when two WO_3-*x*_ CND approach. As a result, the increased concentration of the surfactant tails in the overlap region increases osmotic pressure, generating steric repulsion [25,45]. The theoretical studies indicate the osmotic potential $V_{\mathrm{Osm}}$ is related to the thickness of the polymer layer and the volume fraction of polymer molecules in the layer [46], which is expressed by:

(4) $$V_{\mathrm{Osm}}=0,\;d\geq 2l$$

(5) $$V_{\mathrm{Osm}}=k_{B}T\frac{4\pi a}{v_{1}}\emptyset_{p}^{2}\left(\frac{1}{2}-\chi \right){\left(l-\frac{d}{2}\right)}^{2},\;l\leq d<2l$$

(6) $$V_{\mathrm{Osm}}=k_{B}T\frac{4\pi a}{v_{1}}\emptyset_{p}^{2}\left(\frac{1}{2}-\chi \right)l^{2}\left(\frac{d}{2l}-\frac{1}{4}-\ln\left(\frac{d}{l}\right)\right),\;d<l$$

where a, l, d and $\emptyset_{p}$ represent the radius of a particle, the thickness of polymer layer, the distance between the particles and the effective volume fraction of polymer molecules in the polymer layer, respectively, and $v_{1}$ and χ correspond to the volume of one solvent molecule and the Flory–Huggins solvency parameter, respectively. Here, $\emptyset_{p}$ depends on the concentration of surfactant tails in the overlap region of the surfactant layers [25]. Form Equations (5) and (6), we obtain:

(7) $$\frac{\partial V_{\mathrm{Osm}}}{\partial \left(l/d\right)}=2k_{B}T\frac{4\pi a}{v_{1}}\emptyset_{p}^{2}d^{2}\left(\frac{1}{2}-\chi \right)\left(\frac{l}{d}-\frac{1}{2}\right)>0,\;l\leq d<2l$$

(8) $$\frac{\partial V_{Osm}}{\partial \left(d/l\right)}=k_{B}T\frac{4\pi a}{v_{1}}\emptyset_{p}^{2}\left(\frac{1}{2}-\chi \right)l^{2}\left(\frac{1}{2}-\frac{l}{d}\right)<0,\;d<l$$

According to Equations (7) and (8), the steric repulsion increases with the thickness of the surfactant layers and the number of the surfactant tails in the overlap region. If the surfactant layers are thin, the steric repulsion is increased by increasing the amount of the surfactants.

**Figure 10:**
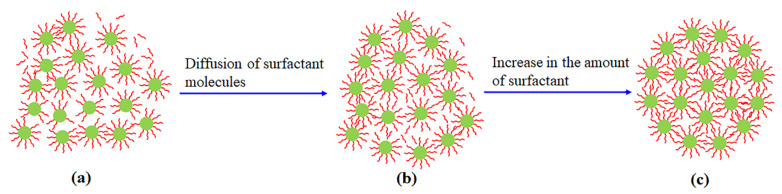
Aggregation schematic of spherical-like and spherical WO_3-*x*_ aggregates. (**a**) irregular structure; (**b**) submicro-spherical-like structure; (**c**) submicro- or micro-spherical structure.

In the initial process of aggregation, the distribution of the WO_3-*x*_ CNDs in the reaction region is nonuniform. Due to long chains of the surfactant molecules, the diffusion is slow. As a result, a number of the WO_3-*x*_ CNDs are partly covered by the surfactant molecules and others are completely capped, which are shown in Figure 10a. Under the attraction interactions, the WO_3-*x*_ CNDs are aggregated in an irregular structure (Figure 10a). With the progress of aggregation, the surfactant molecules gradually come into the irregular structure through diffusion and are adsorbed on the WO_3-*x*_ CNDs (Figure 10b). As shown in Figure 10b, the concentration of the surfactant tails around the WO_3-*x*_ CNDs increases, which leads to the enhancement of the steric repulsion [25]. In this situation, the attraction interactions between the WO_3-*x*_ CNDs are gradually weakened to tune the aggregation process. If the steric repulsion cannot balance the attraction interactions, the WO_3-*x*_ CNDs are aggregated, but the arrangement of the WO_3-*x*_ CNDs is tuned due to the presence of the steric repulsion (Figure 10b). Depending on the steric repulsion, different WO_3-*x*_ CNDs form the submicro-spherical-like or submicro-spherical and micro-shperical structures to reduce the surface energy, which are shown in Figure 10b,c.

Due to the short chain of the butyric acid molecule (~0.6 nm) compared to an oleic acid molecule (~2.5 nm) [10], the butyric acid molecules form thin surfactant layers on the WO_3-*x*_ CNDs. With the increase in the amount of butyric acid, the steric repulsion is enhanced. As a result, the aggregation is further tuned and the structure coverts from the submicro-spherical-like to submicro-spherical shapes (Figure 10b,c). These are the reasons why Figure 5b,c present the submicro-spherical-like and submicro-spherical WO_3-*x*_ aggregates. For oleic acid, the long chain of molecules results in the formation of the thick surfactant layers on the WO_3-*x*_ CNDs, which generates a strong steric repulsion [47]. Furthermore, the separation between the WO_3-*x*_ CNDs is increased. Consequently, the WO_3-*x*_ CNDs are easily rearranged in a large region to form the micro-spherical structure. Therefore, Figure 5d shows the spherical structure with a micron size.

With the increase in the amount of oleic acid in the solution, the viscosity of the solution is enhanced, which leads to a low diffusion speed of the oleic molecules. As a result, there are a small number of the oleic acid molecules to adsorb on the WO_3-*x*_ CNDs. When both the WO_3-*x*_ NPs approach, there are few of the oleic tails between the WO_3-*x*_ CNDs. Based on Equation (5), the steric repulsion is weak, which results in the aggregation of the WO_3-*x*_ CNDs. Simultaneously, some oleic acid molecules adsorbed on the WO_3-*x*_ NPs are deformed by compression during the aggregation [5]. This further shortens the separation between the WO_3-*x*_ NPs and improves the attraction interactions. In this case, the WO_3-*x*_ CNDs are aggregated into the submicro-spherical-like structure, which is confirmed by Figure 5e.

### 3.5. PL Property

**Figure 11:**
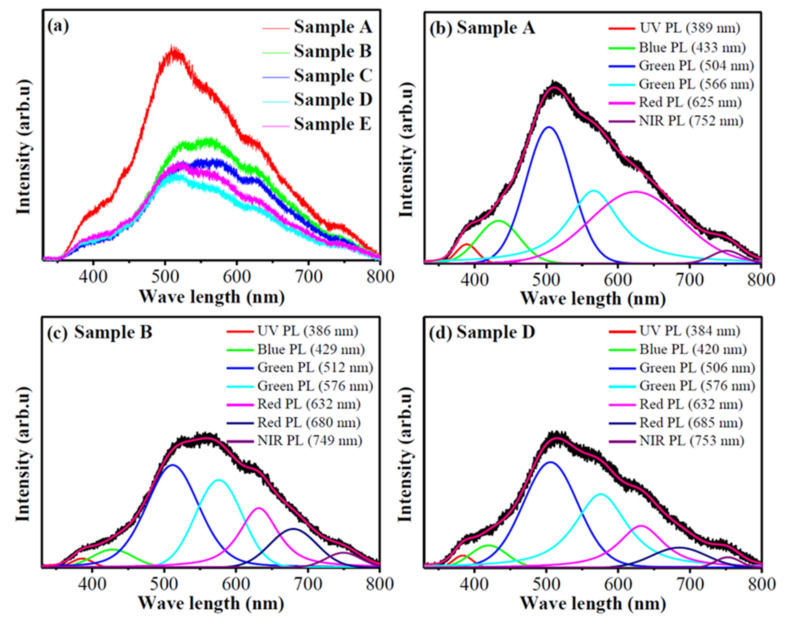
(**a**) PL spectra of samples A–E and (**b**–**d**) deconvoluted PL peaks for the PL spectra of samples A, B and D. The data in the images (**b**–**d**) correspond to the wave lengths of PL emissions.

The PL spectra presented in Figure 11 address the PL properties of the as-synthesized WO_3-*x*_ aggregates. From Figure 11a, one can see that the PL emission from samples B−E exhibits the PL quenching. To confirm the PL bands, a Labspec 5 software was used to fit the PL spectra of samples A, B and D after linear background subtraction. Figure 11a indicates that the PL spectra of samples C and E are similar to the PL spectra of samples B and D, respectively. Here, the PL spectra of samples A, B and D as the typical PL spectra are fitted. The fitted PL peaks are respectively presented in Figure 11b−d, which indicate that the as-synthesized WO_3-*x*_ aggregates emit ultraviolet (UV), blue, green, red and near-infrared (NIR) light.

In the early work [22], the studies on the PL emission from the WO_3-*x*_ nanomaterials indicate that the PL emission is related to the band–band transition and the defect level-level transition. The oxygen vacancies in the WO_3-*x*_ materials form three defect states including the neutral ($V_{O}^{0}$), single charged ($V_{O}^{+}$) and doubly charged ($V_{O}^{2+}$) states [16]. In the electronic structure, the $V_{O}^{0}$ and $V_{O}^{2+}$ states form the resonant state levels in the valence and conduction bands, and the $V_{O}^{+}$ state form a level in the bandgap [16]. Therefore, different transitions give rise to different PL emissions.

According to the early studies [22], the UV emission (384−389 nm) is attributed to the recombination between the electron occupying the resonant defect state in the conduction band and the hole in the valence band, and the blue emission (420−433 nm) can be attributed to the band−band transition. For the transitions related to defect levels, the transition from $V_{O}^{+}$ to $V_{O}^{0}$ leads to the green emission (566−576 nm) and the transitions from $V_{O}^{2+}$ to $V_{O}^{+}$ and $V_{O}^{2+}$ to $V_{O}^{0}$ generate the red emission (625 and 632 nm), respectively. The green emission (504−512 nm) is related to the ${\left(V_{O}^{0}\right)}^{*}$→$V_{O}^{+}$ transition (where * represents the nonrelaxed state). The NIR PL emission (749−753 nm) originates from the relaxation of polarons caused by the coupling of lattice distortion with carries [13].

Compared with Figure 11b, Figure 11c,d exhibit new PL bands at ~680 and 685 nm, which may be related to the coupling between the WO_3-*x*_ CNDs [5]. The aggregation indicates the occurrence of the coupling between the neighboring WO_3-*x*_ CNDs depending on their separation. In the solution containing the surfactant, the short carbon chain (~0.6 nm) of the butyric acid molecule easily causes the coupling between the WO_3-*x*_ CNDs [5,10]. For the oleic acid molecules, the long carbon chain (~2.5 nm) should prevent the coupling [10]. However, their deformation reduces the separation between the WO_3-*x*_ CNDs [5]. As a result, coupling can occur between the WO_3-*x*_ CNDs. The coupling can form a new level in the bandgap of the WO_3-*x*_ materials [5], which generates the PL emission at about 680 and 685 nm.

The PL quenching shown in Figure 11a can originate from the coupling between the WO_3-*x*_ CNDs and the trapping centers arising from the surfactant molecules. Due to the coupling between the WO_3-*x*_ CNDs, the charge transfer occurs between the WO_3-*x*_ CNDs [5], which reduces the recombination probability of the photogenerated electrons and holes to decrease the PL emission. The XPS results indicate that the carboxylic acid molecules coordinate with the WO_3-*x*_ CNDs in bidentate mode. The bidentate structure can trap the photogenerated electrons to quench PL emission [48]. When the PL emission was measured, the irradiation of laser results in the desorption of the residual acetic acid molecules due to small molecular wight, while the butyric and oleic acid molecules are not easily desorbed. As a result, the trapping centers in sample A are reduced and sample A generates strong PL emission (see Figure 11a). Compared with the PL spectra of samples B and D in Figure 11a, the PL intensity of sample D is weaker than that of sample B. This may be related to their aggregation. From Figure 5, one can see that sample D is composed of the micro-spheres, which imply that there are a number of trapping centers. Thus, Figure 11a shows that the PL emission from sample D is weak compared to sample B. Figure 5c,e indicate that there is a small difference in the morphologies of samples C and E, i.e., they may have almost the same amount of trapping centers. Therefore, the PL intensity of sample C is almost the same as that of sample E.

## 4. Conclusions

In summary, the WO_3-*x*_ materials with different structures were synthesized under the butyric or oleic acid surfactants. The results indicate that the WO_3-*x*_ CNDs can be aggregated into the submicro-spherical-like, submicro-spherical or micro-spherical aggregates depending on the steric repulsion of butyric acid or oleic acid, which are related to their amount employed in the process of syntheses. In other words, the increase in the amount of butyric acid leads to the conversion of submicro-spherical-like to submicro-spherical WO_3-*x*_ aggregates, while the increased oleic acid causes the change in the micro-spherical to submicro-spherical-like conversion of the WO_3-*x*_ aggregates. Based on the analyses on the osmotic potential of polymers, the structural conversion of WO_3-*x*_ aggregates originates from the change in the steric repulsion caused by the surfactant layers, the adsorption and deformation of surfactant molecules on the WO_3-*x*_ CNDs. Furthermore, the PL properties of the WO_3-*x*_ aggregates were studied. The WO_3-*x*_ aggregates generate the UV, blue, green, red and NIR PL emission, which are related to the inter-band transition and defect level-associated transition as well as the relaxation of polarons caused by the coupling of lattice distortion with carries. The PL quenching of the submicro-spherical-like, submicro-spherical and micro-spherical WO_3-*x*_ aggregates are associated with the coupling between the WO_3-*x*_ CNDs and the trapping centers originating from the surfactant molecules on the WO_3-*x*_ CNDs. Our achievements can effectively tune the structure of WO_3_-based nanomaterials and contribute to the development of next-generation optoelectronic nanodevices.

## Figures and Tables

**Table 1:** Mass of WCl_6_, volume of carboxylic acids, growth temperature (T), and time (*t*).

Sample	WCl_6_ (g)	Acetic Acid (mL)	Butyric Acid (mL)	Oleic Acid (mL)	T (°C)	*t* (h)
A	2.00	50	-	-	120	12
B	2.00	30	20	-	120	12
C	2.00	20	30	-	120	12
D	2.00	30	-	20	120	12
E	2.00	20	-	30	120	12

**Table 2:** Phases responding to the XRD peaks in Figure 1a,b.

Phase	Peak	JCPDS
A_1_: W_19_O_55_(-105); A_2_: W_19_O_55_(302); A_3_: W_19_O_55_(-2011)	20.5°, 24.0°, 45.9°	45-0167
B_1_: WO_2.92_(-1012); B_2_: WO_2.92_(-1016); B_3_: WO_2.92_(-2128)	18.5°, 24.0°, 49.8°	30-1387
C_1_: W_18_O_49_(-301); C_2_: W_18_O_49_(102); C_3_: W_18_O_49_(103)	14.5°, 16.6°, 24.0°	05-0392
C_4_: W_18_O_49_(302); C_5_: W_18_O_49_(012); C_6_: W_18_O_49_(211)	25.6°, 27.4°, 28.1°	
C_7_: W_18_O_49_(501); C_8_: W_18_O_49_(502); C_9_: W_18_O_49_(-704)	30.6°, 35.8°, 36.6°	
C_10_: W_18_O_49_(021); C_11_: W_18_O_49_(123); C_12_: W_18_O_49_(-523)	49.8°, 54.4°, 55.7°	
C_13_: W_18_O_49_(017); C_14_: W_18_O_49_(-1015)	56.3°, 57.3°	

**Table 3:** Assignment of Raman peaks in Figure 2.

Raman Peak (cm^−1^)	Assignment *	References
196, 198	Lattice vibration	[26]
205, 254	*ν*(W−O−W)	[27]
272, 278, 281	*δ*(O−W−O)	[27,28]
660, 663, 679, 680, 682, 694	*ν*(O−W−O)	[27]
745, 756, 764, 773	*ν*(W−O)	[13,28]
801	*ν*(O−W−O)	[28]
943, 944, 948	*ν*(W=O)	[13,28]

* *δ* and *ν* represent the bending and stretching vibration modes, respectively.

**Table 4:** Binding energy (eV) of deconvoluted XPS peaks in Figure 4.

Sample	W^5+^ 4f_7/2_	W^6+^ 4f_7/2_	W^5+^ 4f_5/2_	W^6+^ 4f_5/2_	O_L_	O_V_	O_C_	(CH_2_)n	CH_2_O	O=C-O
A	34.7	35.5	36.1	37.8	530.4	531.5	532.8	284.7	285.8	289.0
B	34.8	35.6	36.3	37.8	530.4	531.7	533.1	284.7	285.6	288.9
C	34.7	35.6	36.3	37.8	530.4	531.7	533.1	284.7	285.5	288.9
D	34.9	35.6	36.2	37.8	530.5	531.7	533.0	284.7	285.6	288.9
E	34.7	35.6	36.2	37.8	530.3	531.3	532.7	284.7	285.5	288.9

**Table 5:** Areas of O_L_, O_V_ and O_C_ peaks and the area ratio R of O_V_ peak to the area sum of O_L_ and O_V_ peaks.

Sample	O_L_ Peak Area	O_V_ Peak Area	O_C_ Peak Area	Ratio (%)
A	50,970	17,025	11,230	25.0
B	39,685	16,637	12,062	29.5
C	52,013	17,025	10,649	24.7
D	54,684	16,901	11,866	23.6
E	47,576	20,926	14,301	30.5

## Data Availability

Not applicable.
